# Advances in CAR optimization strategies based on CD28

**DOI:** 10.3389/fimmu.2025.1548772

**Published:** 2025-03-13

**Authors:** Sijin Li, Yusi Zhou, Hairong Wang, Gexi Qu, Xuan Zhao, Xu Wang, Rui Hou, Zhangchun Guan, Dan Liu, Junnian Zheng, Ming Shi

**Affiliations:** ^1^ Country Cancer Institute, Xuzhou Medical University, Xuzhou, China; ^2^ Center of Clinical Oncology, The Affiliated Hospital of Xuzhou Medical University, Xuzhou, China; ^3^ Jiangsu Center for the Collaboration and Innovation of Cancer Biotherapy, Xuzhou Medical University, Xuzhou, China; ^4^ College of Pharmacy, Xuzhou Medical University, Xuzhou, China

**Keywords:** CD28, CAR, co-stimulatory molecules, adoptive immune cell therapy, cancer therapy

## Abstract

Chimeric antigen receptor (CAR)-T cell therapy, which utilizes genetic engineering techniques to modify T-cells to achieve specific targeting of cancer cells, has made significant breakthroughs in cancer treatment in recent years. All marketed CAR-T products are second-generation CAR-T cells containing co-stimulatory structural domains, and co-stimulatory molecules are critical for CAR-T cell activation and function. Although CD28-based co-stimulatory molecules have demonstrated potent cytotoxicity in the clinical application of CAR-T cells, they still suffer from high post-treatment relapse rates, poor efficacy durability, and accompanying severe adverse reactions. In recent years, researchers have achieved specific results in enhancing the anti-tumor function of CD28 by mutating its signaling motifs, combining the co-stimulatory structural domains, and modifying other CAR components besides co-stimulation. This paper reviewed the characteristics and roles of CD28 in CAR-T cell-mediated anti-tumor signaling and activation. We explored potential strategies to enhance CAR-T cell efficacy and reduce side effects by optimizing CD28 motifs and CAR structures, aiming to provide a theoretical basis for further clinical CAR-T cell therapy development.

## 1 Introduction

CAR-T therapy has achieved remarkable results in treating hematological tumors and has become a “star therapy” for treating a variety of hematological tumors (1). In addition to the first signal provided by the T-cell receptor (TCR), the activation of T cells requires the activation of co-stimulatory molecules to activate co-stimulatory signals (2, 3). Activation of T cells by signaling through binding CD28 to its ligand is the major co-stimulatory pathway (4–7). The co-stimulatory effect of CD28 depends on its key signaling motifs (8, 9). The activated signaling pathways include the phosphoinositide 3-kinase (PI3K)- protein kinase B (AKT) pathway and the growth factor receptor-bound protein 2 (GRB2)- rat sarcoma virus oncogene (Ras) pathway, which regulate the activation and function of T cells (10, 11). The use of CD28 in CAR structures to provide co-stimulatory signals addressed the problem of the lack of activation of first-generation CAR-T cells. It became one of the most popular and widely used co-stimulatory molecules.

Although CD28-based CAR-T has significantly improved the clinical efficacy of hematological tumors, there are still challenges, such as poor *in vivo* durability and high recurrence rates in patients with hematological tumors and limitations in treatment and safety in solid tumors (12). Resolving these issues is critical to further enhancing CD28-based CAR-T therapies’ efficacy. Currently, optimization strategies for modulating CD28- based CAR-T include altering CD28 intrinsic signaling by mutating CD28 amino acid motifs, exploring combinatorial modes with other co-stimulatory molecules to achieve complementary co-stimulatory signaling, and optimizing CAR structural elements other than the co-stimulatory structural domains, in addition to modulation of the metabolic pathway and the use of artificial intelligence, which have also provided new ideas for optimization. This review summarizes the limitations associated with the application of CD28-based CAR-T cells, evaluates the efficacy of current optimization strategies for CD28-based CAR design, and discusses future perspectives on its clinical and therapeutic potential.

## 2 CD28 signaling function and problems in CAR-T application

### 2.1 CD28 signaling motif and function

**Figure 1:**
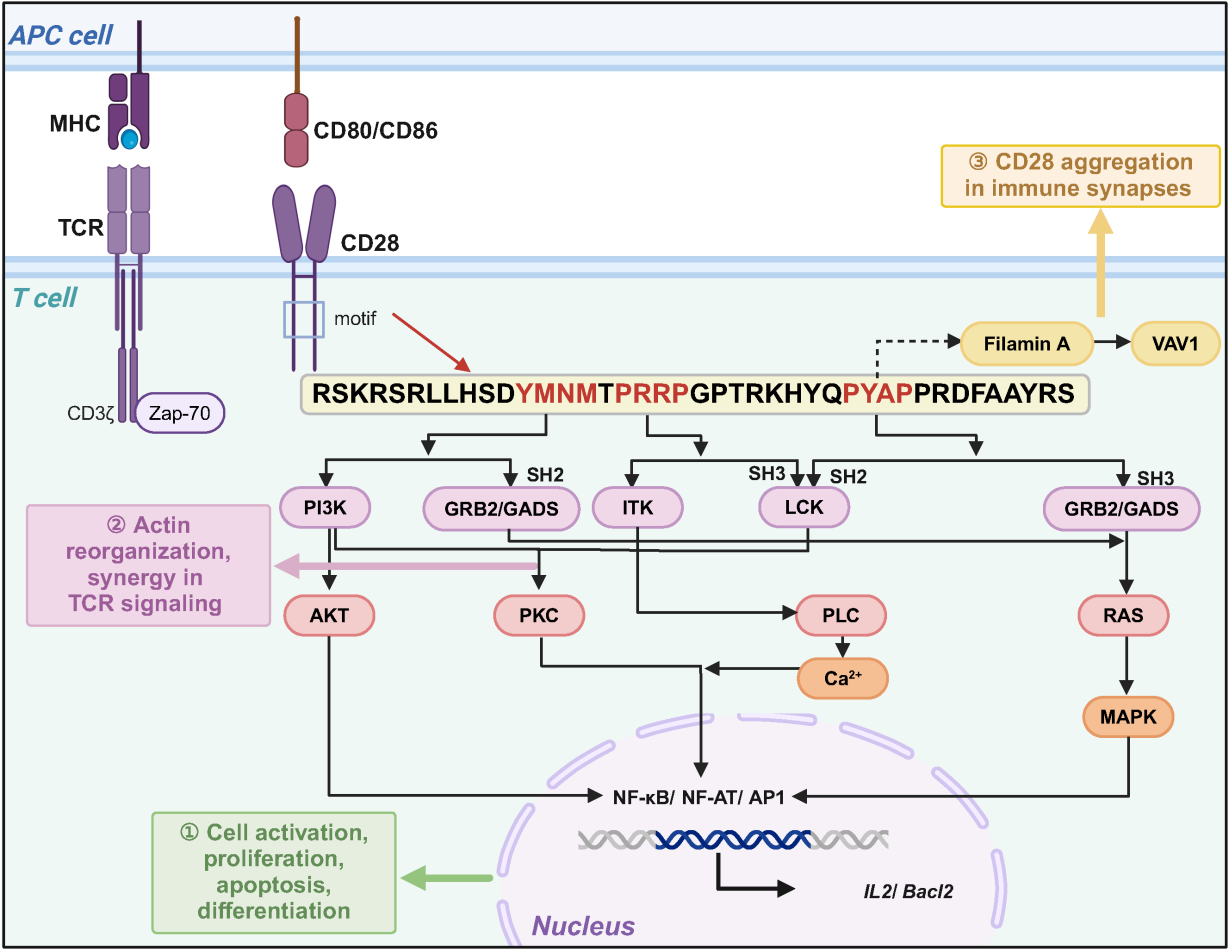
CD28 signaling motifs mediate co-stimulatory signaling to regulate T cell function. After CD28 binds to ligands CD80 or CD86, different signaling motifs activate downstream signaling pathways by binding to specific protein kinases or bridging proteins, which can functionally complement TCR signaling.

Earlier studies have shown that CD28 is expressed in approximately 80% of CD4^+^ T cells and 50% of CD8^+^ T cells (13, 14). It consists of a transmembrane structural domain, an intracellular signaling structural domain, and a variable structural domain-like V-set structural domain connecting the transmembrane structural domain to the signaling structural domain (15). The CD28 signaling structural domain is located in the cytoplasmic structural domain and consists of 41 amino acids, including membrane-proximal YMNM (tyrosine-rich) and proline-rich PRRP and PYAP motifs. These signaling motifs activate downstream signaling pathways by either undergoing phosphorylation or binding to kinases, connexins GRB2 and GADS, and regulate T cell activation, differentiation, cell proliferation, and interleukin-2 (IL-2) secretion (16). For example, upon TCR activation or interaction with the ligand B7 protein, the tyrosine in the YMNM motif undergoes phosphorylation and binds to the p85 subunit of PI3K, which activates PI3K. PI3K can activate the nuclear factor of activated T-cells (NF-AT) transcription factor by activating AKT or regulating protein kinase C (PKC) activity through 3-phosphoinositide-dependent protein kinase-1(PDK-1) (17, 18). PRRP and PYAP bind lymphocyte-specific protein tyrosine kinas (LCK), further activating PKCθ, which shares a downstream signaling pathway with YMNM (19). In addition, PRRP binds to the interleukin-2-inducible T-cell kinase (ITK), which then activates the phospholipase C gamma, (PLCγ), Ca^2+^, and extracellular signal-regulated kinase (ERK)-mediated signaling pathways. For GRB2, GRB2-related adaptor protein downstream of Shc (GADS), YMNM can bind to the SH2 structural domain of GRB2 and activate Ras by bringing son of sevenless (SOS) to the plasma membrane, further activating the downstream mitogen-activated protein kinase (MAPK) pathway (20–25). GADS interacts with SH2 domain-containing leukocyte protein of 76 kDa (SLP-76), an articulatory protein for TCR signaling, through SH3, regulating PLCγ, ERK activation, and Ca2+ pathways, synergistically enhancing NF-AT activity (26–28). However, PRRP and PYAP bind SH3 of GRB2 and GADS and activate downstream signaling pathways. In addition, PYAP recruits and binds Actin protein (Filamin A), which recruits Filamin A to the T-cell membrane, and Filamin A synergistically integrates the signaling pathway with Vav guanine nucleotide exchange factor 1 (VAV1), leading to actin polymerization, mobilization of lipid rafts, and facilitation of CD28 aggregation at the immune synapse (**Figure 1**) (29–32).

### 2.2 Problems with CD28-based CAR-T

**Figure 2:**
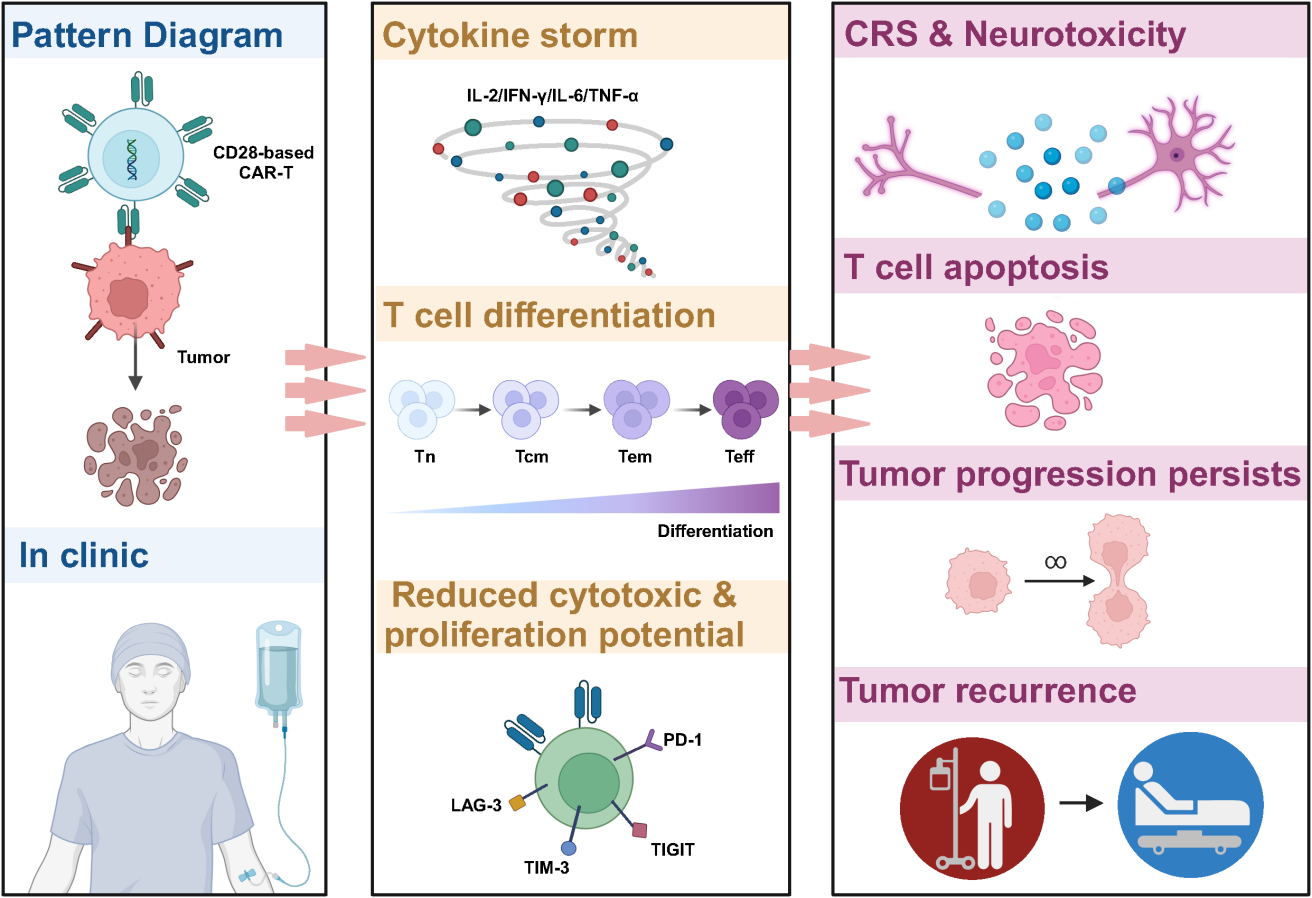
Limitations of CD28-based CAR-T cells in cancer therapy. CD28-based CAR-T cells tend to induce higher levels of cytokine release, triggering CRS and neurotoxicity in patients. In addition, the signaling pathway activated by CD28 leads to reprogramming CAR-T to the glycolysis pathway, which promotes the differentiation of CAR-T to effector T cells and reduces the proportion of memory T cells. This results in poor anti-tumor activity, insufficient persistence *in vivo*, and ineffective resistance to tumor recurrence.

Despite the encouraging results of CD28-based CAR-T in treating hematologic tumors, severe toxic side effects are an essential challenge (**Figure 2**) (33–35). A clinical study in non-Hodgkin’s lymphoma showed that CD28-based CAR-T and 4-1BB-based CAR-T cells exhibited similar anti-tumor effects at 3 months post-treatment, but CD28-based CAR-T cells induced more severe cytokine release syndrome (CRS) and immune effector cell-associated neurotoxicity syndrome (ICANS) (NCT03528421) (36). In addition, in patients with B-cell acute lymphoblastic leukemia (B-ALL) with a high rate of tumor load, CD28-based CAR-T induced a higher incidence of CRS and neurotoxicity, shorter long-term survival, and ineffective resistance to disease recurrence (NCT01044069) (37). Results from clinical trials for relapsed/refractory B-ALL also showed that CD19-CAR-T cells using CD28 as a co-stimulatory molecule triggered a high level of pro-inflammatory cytokine production early after infusion to the patients (NCT01593696, NCT02186860, NCT01044069) (5, 38, 39), which is also consistent with preclinical data on CD28-based CAR-T (40, 41).

The CD28 signaling pathway and its metabolic profile are important factors contributing to the poor persistence of CD28-based CAR-T cells *in vivo*, which often leads to cancer recurrence (37). It has been shown that activation of the PI3K-AKT pathway by CD28 induces an increase in the expression of glucose transporter 1 (Glut1) to promote glucose uptake on the one hand and, on the other hand, enhances the activity of PDK1, which inhibits the decarboxylation of pyruvate and the entry of glucose into the tricarboxylic acid cycle (TCA) cycle, and in turn increase the activity of adenosine triphosphate (ATP)-producing enzymes involved in glycolysis, resulting in cells showing glycolysis-biased metabolic reprogramming (42, 43). The glycolytic pathway promotes the transformation of CAR-T to CD45RO^+^ CCR7^+^ effector memory cells, tending to be short-term effector cells, which, in addition to increasing CAR-T expansion and interferon-gamma (IFN-γ) and IL-2 secretion, often leads to poor CAR-T persistence, which is consistent with the clinical trials showing that *in vivo* survival time after CD28-based CAR-T treatment in patients with hematological tumors tends to be less than three months (44–46). *In vitro* inhibition of PI3K in CD8^+^ T cells delays terminal differentiation, maintains the memory phenotype in CD8^+^ T cells, and may improve the *in vivo* anti-solid tumor therapeutic activity of adoptive CD8^+^ T cells (47). In the CAR-T *in vitro* culture phase, modulating metabolic reprogramming and increasing the percentage of memory cells by pharmacological or gene editing means has yielded promising results in the preclinical study phase, which may provide lessons for solid tumor therapy (48, 49).

## 3 CD28-based CAR structure optimization strategy

### 3.1 CD28 signaling motif optimization

Based on the role of CD28 signaling motifs in mediating CD28 signaling and initiating T-cell function, studies targeting the antitumor effects of CAR-T generated by mutations in the YMNM, PRRP, and PYAP motifs have been conducted (50). In a pancreatic tumor xenograft model, mutation of CD28 YMNM to YMFM in CD28-based CAR-T cells reduces the binding of GRB2 to CD28, decreasing VAV1 signaling, decreasing calcium in-flow, and decreasing NFAT over-activation, thereby decreasing T-cell exhaustion and dysfunction, and increasing the persistence and antitumor efficacy of CAR-T cells in a pancreatic tumor xenograft model (51). Mutating CD28-based CAR-T with PRRP based on mutated YMNM enhances the secretion of IFN-γ and tumor necrosis factor-alpha (TNF-α), reduces the expression of the exhaustion-related transcription factor Nur77, and significantly enhances the cytotoxicity of CAR-T within 48 hours of treatment, demonstrating a superior survival advantage in tumor-bearing mice (52). However, whether CAR-T with mutated YMNM has clinical efficacy needs further exploration.

Moreover, mutating the PYAP signaling motif can potentially enhance CAR-T cell functionality in both hematologic malignancies and solid tumors (53). For example, David M Kofler et al. demonstrated that mutating the PYAPP of CD28 to AYAAA eliminated the ability of PYAP to bind to LCK to eliminate IL-2-induced signaling, reduced the promotion of IL-2 to intratumor regulatory T cell (Treg), and achieved enhanced function of CD28-based CAR-T cells by decreasing the solid Treg cell infiltration in the tumor to achieve the functional enhancement of CD28-based CAR-T, which significantly improved the anti-tumor activity of solid tumors with a large number of Treg infiltration (54). In addition, this PYAP-mutated CD28 construct described above still significantly enhanced T cell proliferation, metabolism, activation, and target cell killing in FAP-targeted CAR-T cells and showed promising efficacy and durability with few side effects in conjunction with a programmed cell death protein 1 (PD-1) blocker in humanized mice suffering from tumors and the first malignant pleural mesothelioma (MPM) patients (55). These studies suggest that CD28-based CAR-T cells with PYAP mutations lacking LCK binding may optimize CAR-T functionality by reducing IL-2-mediated support for Treg cells within the tumor microenvironment, thereby enhancing therapeutic efficacy against solid tumors.

### 3.2 Separating CD28 and CD3ζ signaling

Few tumor-specific antigens (TSAs) and tumor-associated antigens (TAAs) are available for solid tumors, and targeting a single TAA may lead to off-target toxicity of CAR-T to target lowly expressed antigens in healthy tissues (56). Based on this, researchers proposed dual-targeting CAR-T that separates the CD3ζ and the co-stimulatory molecules, which means the CD3ζ-CAR provides suboptimal activation signals upon binding one antigen, and the chimeric co-stimulatory structural domain containing the co-stimulatory receptor provides T-cell co-stimulatory signals upon recognition of the second antigen (57). This design enhances the responsiveness of CAR-T cells to double-positive tumor cells and reduces the risk of off-targeting and tumor antigen escape. For example, Evripidis Lanitis developed the mesothelin (Meso) single chain variable fragment (scFv)-CD3ζ and folate receptor alpha (FRα) scFv-CD28-based CAR, which had no potent activity against normal tissues expressing only mesothelin; meanwhile, they showed potent anti-solid tumor activity and persistence *in vivo* (58). Another study of the glypican 3 (GPC3)-CD3ζ and asialoglycoprotein receptor 1 (ASGR1)-CD28-41BB- against hepatocellular carcinoma (HCC) CAR-T cells study also demonstrated that CAR-T cells separating CD28 and CD3ζ signals had potent antitumor activity and safety against tumor cells carrying both antigens. However, the design depends on recognizing the two antigens, and its complex engineering may increase the production cost. Its effectiveness in solid tumor microenvironment (TME) still needs to be further explored, and the discovery of novel tumor antigens may promote the application of this strategy in solid tumors.

### 3.3 Combination of CD28 and other co-stimulatory molecules

**Figure 3:**
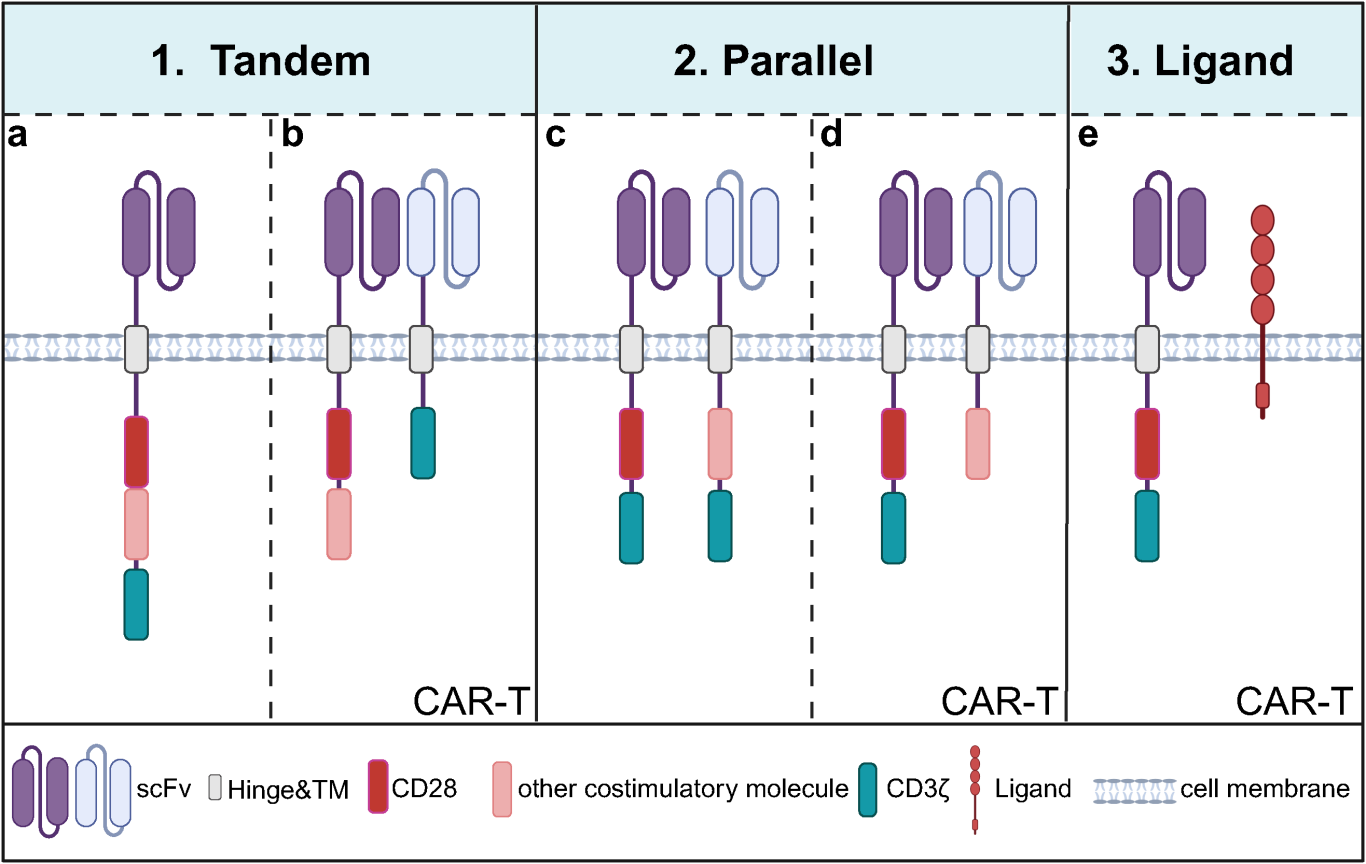
Current modes of combining co-stimulatory molecules of CARs containing CD28. **(a)** A common tandem mode of co-stimulatory molecule involves linearly fusing CD28 with other costimulatory molecules, such as 4-1BB or OX40, within a CAR that targets a single antigen; **(b)** Another tandem mode entails T cells carrying two CARs: one CAR contains two linearly fused co-stimulatory molecules but lacks the CD3ζ domain, while the other is a first-generation CAR targeting a different antigen utilizing the CD3ζ domain for intracellular signaling but without costimulatory molecules; **(c)** One of the co-stimulatory molecules in parallel mode involves T cells carrying two second-generation CAR that recognizes the two antigens; **(d)** Another parallel mode consists of T cells expressing a second-generation CAR with CD28 while simultaneously expressing another CAR that lacks the CD3ζ domain but recognizes a different antigen; **(e)** T cells express a second- or third-generation CAR containing CD28 and simultaneously express a ligand for other co-stimulatory molecules.

Second-generation CARs, with the addition of co-stimulatory molecules, have significantly enhanced in mediating T cell proliferation, cytokine release, and *in vivo* antitumor function compared with first-generation CARs, illustrating the importance of co-stimulatory molecules. However, different co-stimulatory molecules have advantages and disadvantages in cell activation, cell differentiation, proliferation, metabolism, and *in vivo* safety (59). Based on this, researchers have proposed combining different costimulatory molecules to achieve complementary benefits (**Figure 3** and **Table 1**).

**Table 1:** Optimization strategies for combinatorial modes targeting CD28 with other co-stimulatory molecules.

Combined model	Co-stimulatory molecules/ligand	Target	Tumor	Reference
Tandem	CD28、4-1BB	CD19	leukemia	(53)
		CD19、PSMA	prostate cancer	(57)
		Glypican-3	liver cancer	(63)
		PSMA	prostate cancer	(64)
		Glypican-3、ASGR1	liver cancer	(111)
		PSMA	prostate cancer	(112)
		CD19、CD20	lymphoma	(113)
		CEA	Colorectal Cancer	(68)
		GD2	Neuroblastoma	(114)
		BCMA、TACI	Multiple Myeloma	(115)
	CD28、4-1BB、CD27	BCMA	Multiple Myeloma	(66)
	CD28、OX40/4-1BB	CD30	Lymphoma	(69)
		LMP1	Nasopharyngeal Cancer	(70)
	ICOS、4-1BB	MSLN	Solid tumor	(71)
	CD28、CD40	CD19	B-cell lymphoblastic leukemia, B-cell non-Hodgkin's lymphoma	(116)
Parallel	CD28、4-1BB	CD19	Leukemia	(74)
		BCMA、CD19、CD38	Multiple myeloma, acute lymphoblastic leukemia	(75)
		GD2、B7H3、MSLN、CSPG4	Neuroblastoma	(76)
		BCMA、GPRC5D	Multiple Myeloma	(77)
		M-CSFR、IL34	Lymphoma, Breast	(117)
		CD19、CD20	Lymphoma	(118)
		BAFF-R、APRIL	Multiple Myeloma	(119)
Ligand-based	CD28、4-1BB/ICOSL	GPC3	Liver Cancer	(78)
	CD28/4-1BBL	CD19	Acute Lymphoblastic Leukemia	(79)
		CD19	Relapsed Refractory Lymphoma	(80)
		B7H3	Solid tumor	(81)
	CD28/CD40L	CD19	Chronic lymphocytic leukemia	(82)
		CD19	Leukemia、Lymphoma	(83)

#### 3.3.1 Tandem mode

The tandem mode of co-stimulatory molecules is characterized by constructing two or more co-stimulatory molecules in a single CAR by tandem mode. The most studied mode is the tandem combination of CD28 and 4-1BB (60). A CAR containing CD28 triggers rapid and high-intensity lysis of target cells, while a CAR containing 4-1BB triggers a low-intensity and long-lasting response (61, 62). However, preclinical studies of CAR-T cells tandeming CD28 and 4-1BB have shown that the effect of tandem combination is not a simple signaling superposition. The effect of tandeming is related to both tumor type and programmed death-ligand 1 (PD-L1) expression level (63–65). For example, tandem mode in combination with blockade of PD-L1/PD-1 or secretion of anti-PD-L1 scFv enhances the killing activity of tumor cells targeting high levels of PD-L1. It reduces the exhaustion of CAR-T cells (66). In addition, other co-stimulatory molecules tandemly associated with CD28 are OX40 and inducible T-cell costimulator (ICOS) (67). CAR-T cells containing tandem CD28-OX40 showed significantly higher T-cell expansion and IL-2 secretion levels than CD28-based CAR-T cells. The mechanism is that OX40 signaling inhibits the secretion of IL-10 by CAR-T cells and reduces the inhibition of other T-cell functions in the TME (68). However, comparing the antitumor activity of the tandem mode of CD28-4-1BB versus CD28-OX40 has shown inconsistent results in different studies (69, 70). ICOS is a co-stimulatory molecule belonging to the CD28 family, and the CAR-T antitumor activity of ICOS in tandem with other co-stimulatory molecules may be related to other structures in the CAR. For example, studies have shown that CD28 in tandem with ICOS significantly enhances the persistence of CD8^+^ CAR-T cells, and ICOS and 4-1BB tandem have an antitumor advantage in solid tumors (71, 72). ICOS-OX40 tandem maintains high target cytolysis toxicity despite multiple rounds of tumor stimulation *in vitro* (73). Overall, the combination of co-stimulatory molecules targeting CD28 is influenced by tumor type, tumor microenvironment, and CAR structure, and the optimal mode of co-stimulatory molecule tandem will need to be determined in the future based on specific tumors.

#### 3.3.2 Parallel mode

Parallel mode refers to the mode of CAR carrying two different co-stimulatory molecules in a single T cell (74, 75). Studies have shown that”two co-stimulatory molecules sharing one CD3ζ” has better antitumor efficacy than the parallel mode of “two co-stimulatory molecules sharing two CD3ζs”. Separate activation of CD3ζ by dual-targeted CAR resulted in the overactivation of CAR-T cells and induced cellular exhaustion, possibly a reason for its poor effectiveness (76, 77). In addition, for the parallel mode of CD28 and 4-1BB, it was demonstrated that dual-targeted CAR-T carrying CD28 and 4-1BB sharing one CD3ζ had higher tumor-killing activity, dividing and proliferating ability and durability than the parallel mode of dual co-stimulatory molecules sharing two CD3ζ (76). The problem with this model is that if two individual CARs are transduced into T cells, it cannot be guaranteed that both CARs are expressed at the same level in all the transduced cells, and if it is a single double cis-trans vector containing two individual CARs, the transduction efficiency of the double CAR may be affected due to the large size of the vector. Moreover, the current co-stimulatory molecules in parallel combination are only CD28 and 4-1BB, and the antitumor potential of other co-stimulatory molecules in parallel combination remains to be explored.

#### 3.3.3 Combined patterns of co-stimulatory molecules and ligands

In addition, simultaneously expressing a co-stimulatory molecule with a ligand for another co-stimulatory molecule in T cells can generate synergistic co-stimulatory signaling (78). For example, the combination of CD28-4-1BBL can continuously complement 4-1BB signaling after CD28 signaling activation. The concurrently activated interferon regulatory factor 7 (IRF7)/IFNβ signaling pathway initiates DCs in the TME and inhibits Treg activation. CD28-4-1BBL-based CAR-T has shown safe and effective results in targeting CD19 hematologic tumors in both preclinical and clinical trials (NCT03085173) (79, 80), and even effective anti-tumor effects in solid tumors targeting B7-H3 (81). These results illustrate that the combination of CD28 and 4-1BBL can spatiotemporally and spatially differentially activate both CD28 and 4-1BB co-stimulatory signaling and that this synergistic co-stimulatory signaling may surpass CAR target antigens and activate the host immune system, which provides a new strategy for modulating TME to enhance CAR-T function. Based on the role of the CD40 pathway in improving anti-tumor response, some researchers combined CD28 with CD40L in CD19 CAR-T, which up-regulate the proliferation of T cells, the expression of CD80 and CD40 of dendritic cells after co-culture with CD40-positive tumor cells, increase the recruitment of endogenous effector T cells by DC, and enhance the immunogenicity of CD40-expressing tumor cells. However, clinical safety needs to be investigated as the CD40L/CD40 pathway may induce a systemic inflammatory response (82, 83).

### 3.4 Optimization of CD28 upstream and downstream structural domains

Single-chain variable fragments are the antigen-binding domains of CAR structures, and it has been shown that lowering the affinity of scFV for antigen binding can reduce reactivity to antigens expressed at physiological levels while maintaining potent antitumor activity, thereby attenuating off-target toxicity (84). Common CAR hinge domains/spacer regions consist of the IgG1 or IgG4 hinge regions and the CH2-CH3 structural domains of immunoglobulin G fragment crystallizable (IgG Fc). The length and composition of the hinge domain is an important factor in designing CARs with optimal antitumor activity or low toxicity (85–87). Although no reports show the relationship between CD28 and the function of the scFV and hinge region, CAR is a combinatorial entity, and exploring CD28 optimization strategies for specific scFV and hinge region patterns is expected to improve the application of CAR-T. The transmembrane structural domain anchors CAR to the T cell membrane and transduces ligand recognition signals to the cytoplasmic signals of the TCR, which plays a critical role in CAR expression or structural stability (72). Studies have shown that both extracellular and transmembrane structural domains of CD28 can partially induce T cell activation (88), providing more choices of CAR transmembrane domains. CD3ζ instead of FcRγ has been widely used as a CAR signaling structural domain (89). The cytoplasmic tail of CD3ζ contains three immunoreceptor tyrosine-based activation motifs (ITAMs), and CD28-based CAR studies have shown that the number and position of ITAMs are related to CAR-T cell function (90, 91). For example, retaining only the second position of ITAMs in CD3ζ reduces apoptosis of CD28-based CAR-T cells that target ErbB2 (92). For CD19-CAR, placing ITAM1 or ITAM3 of CD3ζ in CD28-based CAR close to the proximal membrane position of CARs targeting CD19 or inserting an IL-2Rβ structural domain between CD28 and CD3ζ, and inserting a YXXQ motif in the distal region of the CD3ζ structural domain can improve the persistence and therapeutic efficacy of CAR-T cells (2, 93). In summary, the scFV, hinge region, transmembrane region, and signal transduction region can be comprehensively optimized in combination with tumor specificity and individual patient differences to develop a CD28-based CAR with the best overall performance.

## 4 Conclusion and prospective

**Figure 4:**
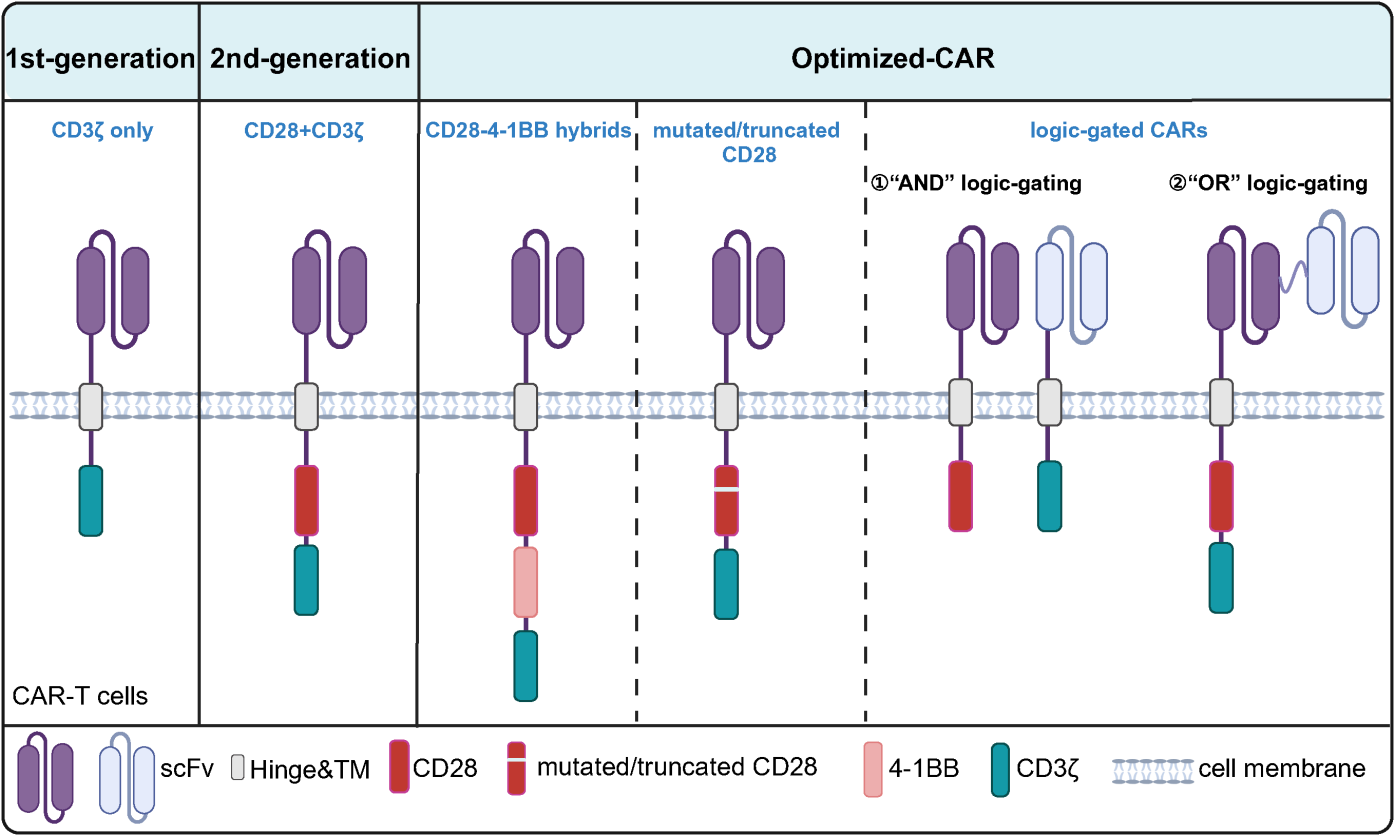
Structural Evolution of CD28-Based CARs. The first-generation CARs contain only the CD3ζ chain as the signaling domain. The second-generation CARs, built upon the first-generation design, incorporate CD28 as a co-stimulatory domain. Further optimization of CD28-based CARs includes combinations of CD28 with other co-stimulatory molecules such as 4-1BB, as well as mutated/truncated CD28 and "AND" or "OR" logic-gated CARs.

**Figure 5:**
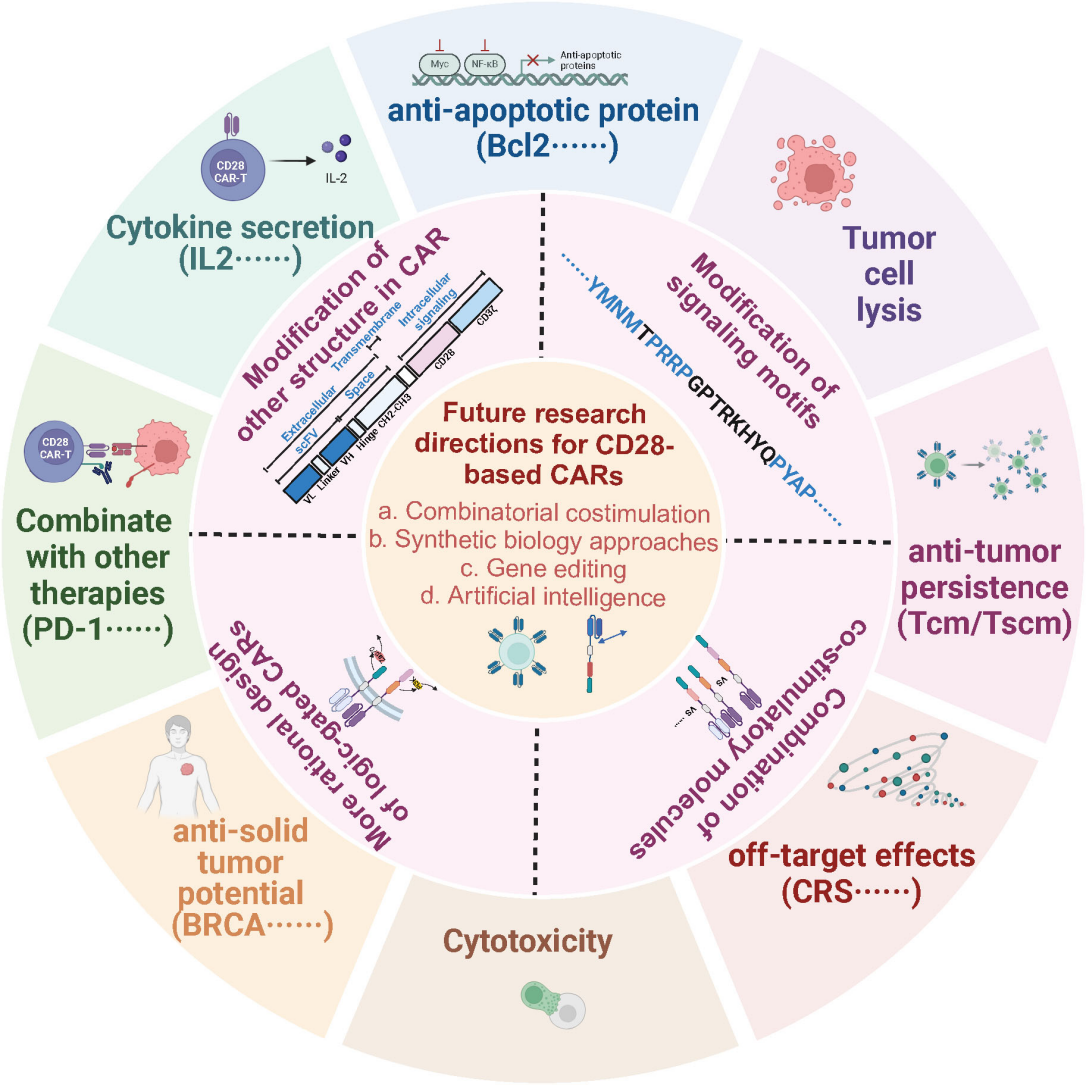
Optimization schemes for CD28-based CAR structures. Strategies to optimize the CD28-based CAR structure include mutating the signaling motif of CD28, combining it with other costimulatory molecules, separating the CD3ζ and CD28 signaling motifs or optimizing other modules in the CAR structure to regulate the activation, differentiation, survival, proliferation, and antitumor activity of CAR-T cells to enhance the durability and antitumor efficacy of CAR-T cell therapy, which offers potential to expand the application of CAR -T therapy in hematological or solid tumor applications.

CD28 has been widely used in preclinical and clinical studies (**Figure 4**). CAR-T cells with CD28 as a co-stimulatory structure suffer from rapid exhaustion and poor durability in therapy, accompanied by more significant adverse effects and higher relapse rates. Studies have shown that optimizing the CD28-based CAR structure—through approaches such as mutating the CD28 signaling motif, combining it with other co-stimulatory molecules, and modifying its upstream and downstream structures—is expected to improve the durability and anti-tumor efficacy of CAR-T cells by enhancing their ability to regulate T cell proliferation and survival, with a relatively optimistic safety profile (**Figure 5**, **Table 2**). Currently, the existing CD28 modifications have been successful, but CD28-based CAR-T cells face much greater complexity in real human tumor environments compared to *in vitro* or mouse tumor models. Moving forward, there is a need to develop more accurate CAR-T evaluation models to assess the anti-tumor efficacy of CD28-modified CAR-T cells, including metrics such as tumor-killing ability, differentiation, persistence, and exhaustion. In the future, leveraging the latest advancements in synthetic biology and gene editing technologies will help optimize logic-gate CARs and develop more rational combinations of CD28 mutations or co-stimulatory molecules.

**Table 2:** The CRS and toxicity levels of CD28-based CARs following structure optimization in clinical trials.

Study (year of publication)	Patient population (n)	Target	Costimulatory domain	CR	CRS rate; grade≥3 CRS rate	Neurotoxicity rate; grade≥3 neurotoxicity rate
Lujia Dong et al. (2015) (120)	B-ALL (50)	CD19	CD28/4-1BB/CD27	86%	94%; 16%	not mentioned
LungJi Chang et al. (2016) (121)	B-ALL (102)	CD19	CD28/4-1BB/CD27	86.3%	71.6%; 10.8%	not mentioned
Andras Heczey et al. (2017) (122)	R/R NB (11)	GD2	CD28/OX40	18.2%	9.1%; 0%	90.9%; 0%
Gunilla Enblad et al. (2018) (123)	BCL (11), ALL (4)	CD19	CD28/4-1BB	40%	20%; 6.7%	6.7%; 13.3%
Carlos A et al. (2018) (124)	R/R NHL (16)	CD19	CD28/4-1BB	18.8%	40%; 0%	6.3%; 6.3%
Jae H. Park et al. (2018) (80)	R/R CLL (9), DLBCL (6), tFL (3), FL and WM (4), RT (3)	CD19	CD28/4-1BBL	57%	67%; 0%	33%; 8%
Xuan Zhou et al. (2020) (125)	R/R NHL (21)	CD19	CD28/CD27	43%	14%; 0%	4.8%; 0%
Cheng Jiao et al. (2021) (126)	R/R BCL (4)	CD19、CD22、CD30、GD2、PSMA	CD28/CD27	25%	50%; 0%	0%; 0%
Hui Liu et al. (2021) (127)	R/R NHL (17)	CD19	PD-1/CD28	41%	88.3%; 0%	0%; 0%
Lihua Yu et al. (2021) (128)	R/R NB (10)	GD2	CD28/4-1BB	0%	90%; 0%	0%; 0%
Zhuohao Liu et al. (2023) (129)	GBM (8)	GD2	CD28/4-1BB	0%	0%; 0%	0%; 0%
Patrick Derigs et al. (2024) (130)	R/R ALL, CLL, BCL (9)	CD19	CD28/4-1BB	66.7%	77.8%; 11%	0%; 0%
Tessa Gargett et al. (2024) (131)	mutant metastatic melanoma, solid tumors (12)	GD2	CD28/OX40	0%	8.3%; 0%	0%; 0%

CAR optimization strategies based on mutated CD28 signaling motifs indicate that CD28 participates in multiple signaling pathways. Selective manipulation of these pathways can achieve long-term persistence and anti-tumor activity of CAR-T cells, which is attributed to the application of gene editing technology in CAR structure design, which is expected to provide further space for mutation or modification of CD28 structural domains. Various combination modes of CD28 with other co-stimulatory molecules have been developed, demonstrating promising effects by synergizing multiple co-stimulatory signals. Multiple co-stimulatory signals have mobilized the host’s immune response by complementing each other’s strengths and even modulating dendritic cells (DCs), Treg cells, and immunosuppressive PD-L1/PD-1 in TMEs, increasing the potential of CAR-T therapies to be applied in solid tumors.

An important reason for the poor treatment of CAR-T solid tumors is the presence of multiple metabolic inhibitors (lactate, reactive oxygen species, prostaglandin E2) in the TME (94–97), which severely impairs CAR-T cells’ antitumor activity. Strategies to overcome this obstacle are mainly to express relevant molecules or enzymes resistant to metabolic inhibitors in TME (catalase) on CAR-T cells through gene editing (98, 99) or to express genes that enhance mitochondrial biogenesis and function (PGC1α) (100). Current modification strategies engineering CD28-based CAR-T resistance to TME are primarily limited to incorporating TME-resistant response elements, attenuation of PD-L1/PD-1 inhibitory signaling, and combination of metabolic regulatory drugs to orchestrate CD28 signaling to enhance CAR-T cell activity, and no modification of CD28 signaling or sequence has been reported. In the future, exploring the antitumor efficacy of resistance to TME triggered by CD28 mutation strategies in the construction of ex vivo TME models that mimic the hypoxic, high-lactate, glucose-competitive, and immunosuppressive features of TME (101, 102) could further develop TME-induced enhanced CD28, or design response elements targeting the inhibitory signaling on the basis thereof, offering the possibility of CAR-T therapy for broader application to cancer and improved efficacy.

Combining metabolic regulation with strategies to increase resistance to the tumor microenvironment can effectively enhance CAR-T cells’ survival and anti-tumor ability in the tumor microenvironment. A deeper understanding of the mechanisms of metabolic regulation can help to tailor tumor immunotherapy regimens to individual patients. For instance, considering the differences in the tumor microenvironment and T-cell status of different patients and selecting the most suitable CD28-based CAR design and metabolic regulation strategies to improve therapeutic efficacy and reduce adverse effects, may offer hope for CAR-T precision therapy. However, we must also be concerned about the possible toxicity risks of adding multiple co-stimulatory molecules. For example, a clinical trial treating 11 patients with non-Hodgkin’s lymphoma showed that tandem of a Toll-like receptor 2 (TLR2) TIR structural domain in a CD28-based CAR structure targeting CD19 resulted in severe CRS in 2 patients (18%) and severe ICANS in 1 patient (9%) (NCT04049513) (103). With the help of new technological tools, dynamic regulation of CD28 and other signaling intensities might provide safety for the combination of co-stimulatory molecules.

Currently, the application of artificial intelligence (AI) in CAR-T is at the frontier of exploration. The application of AI in CAR-T cancer therapy mainly focuses on AI-assisted prognosis of clinical efficacy of patients (104), establishment of toxicity prediction models, and assessment of side effects after CAR-T treatment (105, 106). Studies directly applying AI technology to optimize the structure of CARs have only focused on designing antibodies against cancer targets by using the protein design tool RFdiffusion to create antibodies against cancer targets (107) or using AI to design protein conjugates with high affinity for cancer antigens to replace scFV (108). AI has accelerated the development of CAR-T products by designing candidate proteins for binding to the target antigens of CAR-T. However, whether the antibodies or protein conjugates can be safely applied to humans without inducing immune responses requires further validation through experiments. In addition, for optimizing CAR signaling, including prediction and optimization of CAR-T tonic signaling using CAR-Toner’s AI tools (109), prediction of unnatural combinations of specific signaling motifs combinations and configurations affecting T-cell phenotypes (110). However, AI-based prediction of optimized CD28 signaling has not yet been reported. In the future, perhaps it may be possible to use the AI algorithms to construct virtual models for predicting CAR-T functional effects due to mutated CD28 signaling sites, accelerating the understanding of CD28 signaling mechanisms, based on which, combined with existing databases, may be able to provide personalized CD28-based CAR optimization solutions for cancer patients. Through AI’s learning of large amounts of data and the development of additional AI algorithms, it may be possible to predict the effects of different molecular combinations or modifications on CD28 co-stimulatory activity and further expand to predict the efficacy of changes including modification of scFV, hinge region, transmembrane region, and CD3ζ, which is expected to provide a basis for optimizing CAR-T cell therapy.
